# Recent advances in understanding carotenoid-derived signaling molecules in regulating plant growth and development

**DOI:** 10.3389/fpls.2015.00790

**Published:** 2015-09-24

**Authors:** Li Tian

**Affiliations:** Department of Plant Sciences, University of California, Davis, DavisCA, USA

**Keywords:** apocarotenoid, carotenoid, chloroplast, development, retrograde signaling, root, shoot, signaling

## Abstract

Carotenoids (C_40_) are synthesized in plastids and perform numerous important functions in these organelles. In addition, carotenoids can be processed into smaller signaling molecules that regulate various phases of the plant’s life cycle. Besides the relatively well-studied phytohormones abscisic acid (ABA) and strigolactones (SLs), additional carotenoid-derived signaling molecules have been discovered and shown to regulate plant growth and development. As a few excellent reviews summarized recent research on ABA and SLs, this mini review will focus on progress made on identification and characterization of the emerging carotenoid-derived signals. Overall, a better understanding of carotenoid-derived signaling molecules has immediate applications in improving plant biomass production which in turn will have far reaching impacts on providing food, feed, and fuel for the growing world population.

## Carotenoid-derived Signals in Shoot and Root Development

**FIGURE 1:**
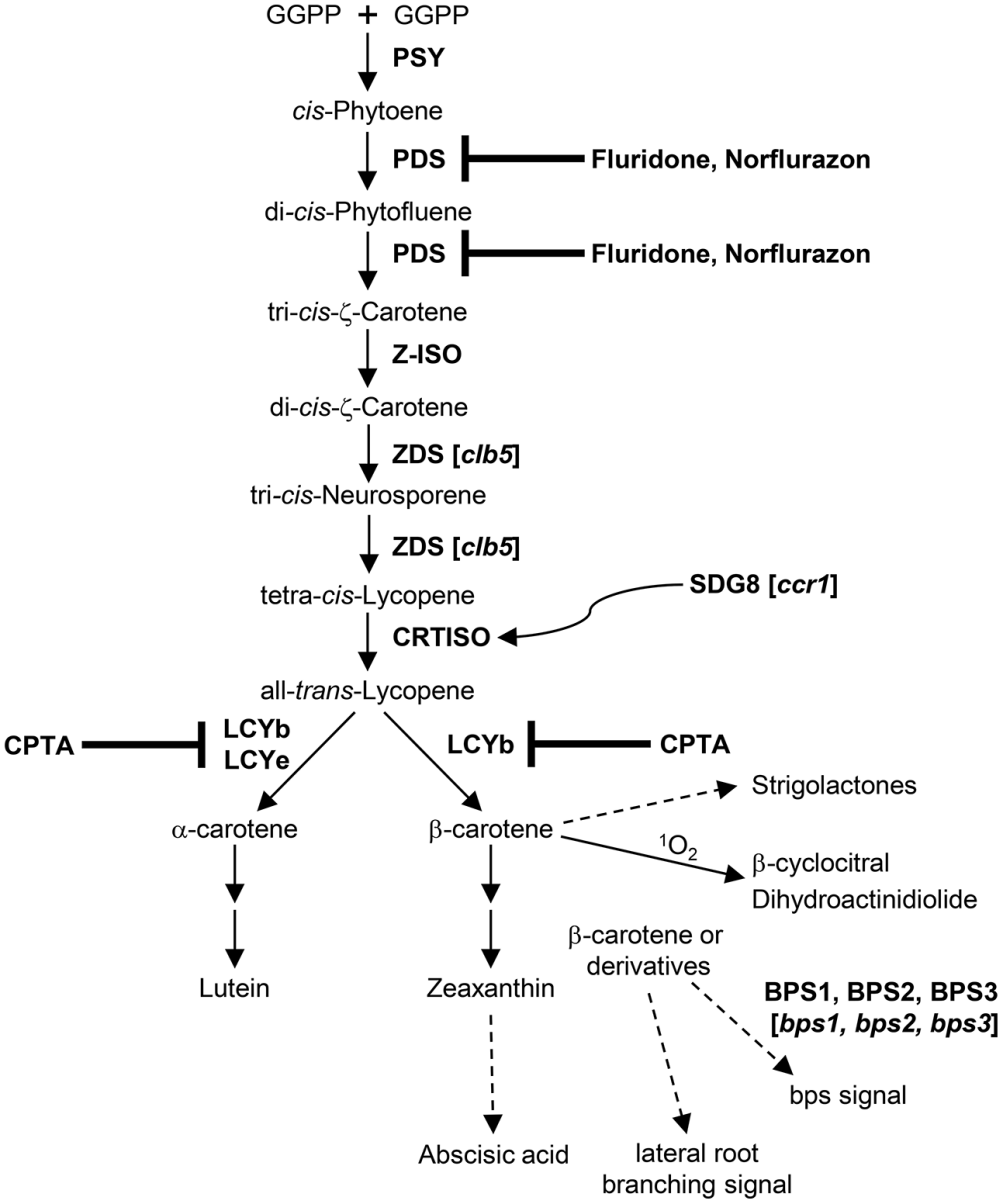
**Biogenetic relationships of carotenoid-derived signal molecules in *Arabidopsis*.** Steps blocked by herbicides are indicated. Dotted arrows represent multiple reactions or unknown processes. A curved arrow indicates a regulatory relationship, not a direct chemical reaction. Mutations of the corresponding biosynthetic or regulatory genes are shown in brackets. GGPP, geranylgeranyl pyrophosphate; PSY, phytoene synthase; PDS, phytoene desaturase; Z-ISO, ζ-carotene isomerase; ZDS, ζ-carotene desaturase; CRTISO, carotenoid isomerase; LCYb, lycopene β-cyclase; LCYe, lycopene ε-cyclase; CPTA, 2-(4-chlorophenyl-thio) trimethylamine hydrochloride; BPS1 (2,3), BYPASS1 (2,3); *bps1* (*2,3*), *bypass1* (*2,3*); *ccr1, carotenoid chloroplast regulatory1*; *clb5, chloroplast biogenesis5*; SDG8, Set Domain Group8.

About a decade ago, allelic *bypass1* (*bps1*) mutants that lack leaf vasculature and trichomes, produce short roots and exhibit arrested shoot apical meristem (SAM) activities were isolated from ethyl methanesulfonate (EMS) mutagenized *Arabidopsis* grown at 16°C (Van Norman et al., 2004). The shoot and root phenotypes of *bps1* were alleviated when the mutant plants were grown at higher temperatures (Van Norman et al., 2004). Through elegant genetic and biochemical analyses of the *bps1* mutant and wild type plants, the Sieburth group showed that a mobile bypass (bps) signal is generated in roots of wild type *Arabidopsis* plants, which regulates root and shoot development through root-to-shoot signal transmission (Van Norman et al., 2004). Since application of carotenoid biosynthesis inhibitors or introduction of the *pds1* mutation (containing an impaired carotenoid biosynthetic enzyme phytoene desaturase/PDS) could partially rescue the abnormal *bps1* growth phenotype, it was hypothesized that bps could be a signal originated from carotenoids (Van Norman et al., 2004; Van Norman and Sieburth, 2007). Further molecular, biochemical and genetic examinations indicated that the bps signal is distinct from ABA and SLs; its production entails synthesis of β-carotene branch carotenoids but does not require the activity of a single carotenoid cleavage enzyme (9-*cis*-epoxycarotenoid dioxygenase/NCED or carotenoid cleavage dioxygenase/CCD) (Van Norman and Sieburth, 2007; **Figure 1**). Besides *BYPASS1* (*BPS1*), two additional *BPS* genes (*BPS2* and *BPS3*) were identified in *Arabidopsis* and the *bps* triple mutant (*bps1bps2bps3*) has aberrant cell divisions during early embryogenesis that result in defective SAM, root apical meristem (RAM), and vascular meristem (VM) (Lee et al., 2012). Such embryonic defects are not evident in *bps1* and less pronounced in the *bps1bps2* double mutant, suggesting that the three *Arabidopsis BPS* genes possess overlapping yet distinct functions in generating the bps signal (Lee et al., 2012). Analyses of the auxin response markers as well as localization and trafficking of the PIN1 auxin eﬄux transporter in *bps* mutants revealed that the auxin signaling pathway is not a primary target of the bps signal for regulation of plant development (Lee and Sieburth, 2012; Lee et al., 2012). Though the chemical structure of bps is currently unknown, progress has been made recently toward elucidation of the bps signal using a bioassay-based purification and identification scheme (Adhikari et al., 2013).

Another shoot development mutant, *carotenoid chloroplast regulatory1* (*ccr1*), was identified by the Pogson group in a screen for *Arabidopsis* mutants defective in carotenoid and chloroplast regulation (Cazzonelli et al., 2009). Unlike *bps1* that contains a lesion in a gene of unknown function, *ccr1* encodes a malfunctional histone methyltransferase Set Domain Group8 (SDG8) (Cazzonelli et al., 2009; **Figure 1**). As a result of the *ccr1* mutation, histone methylation of chromatin around carotenoid isomerase (CRTISO, an enzyme that converts tetra-*cis*-lycopene to all-*trans*-lycopene) and *CRTISO* expression were reduced, which led to significantly decreased accumulation of lutein and increased levels of β-carotene branch xanthophylls (Park et al., 2002). The *ccr1* mutant exhibited more abundant rosette and cauline branching than wild type plants, yet, unlike the *Arabidopsis* shoot branching mutants deficient in SLs, the *ccr1* mutant phenotype could not be suppressed by a root generated, transmissible signal when grafted to the wild type *Arabidopsis* root stock (Cazzonelli et al., 2009). Though changes in carotenoid content were observed in *ccr1*, it remains to be elucidated whether increased shoot branching in this mutant is indeed associated with a carotenoid-derived signal.

In addition to mutant analysis (e.g., *bps1, ccr1*) that allows efficient identification of altered plant growth and the corresponding causal mutations, chemical treatment of wild type *Arabidopsis* seedlings has also been valuable in uncovering a carotenoid-derived signal that controls lateral root (LR) branching. Van Norman et al. (2014) measured the root’s capacity for LR formation (i.e., LR capacity) based on the number of prebranch sites and observed a decrease in LR capacity in *Arabidopsis* seedlings treated with carotenoid biosynthesis inhibitors, norflurazon (NF) and 2-(4-chlorophenyl-thio) trimethylamine hydrochloride (CPTA). Further support for association of reduced LR capacity with carotenoid deficiency was provided by application of NF and CPTA to *Arabidopsis* expressing a transcriptional reporter of auxin response, *pDR5:LUC*; it was shown that inhibition of carotenoid production disturbed the rhythmic oscillation of the LR clock necessary for establishment of prebranch sites (Van Norman et al., 2014). Since ABA or SL deficiency does not impose a similar impact on LR capacity as carotenoid biosynthesis inhibitor treatment, a novel carotenoid-derived signal in LR determination and development has been proposed. Generation of this signal appears to require a CCD activity as treatment of *Arabidopsis* seedlings with a CCD inhibitor D15 led to significantly decreased LR capacity (Van Norman et al., 2014). Since D15 treatment, which blocks CCD activities and prevents carotenoid turnover, caused substantially more increases in root carotenoid content than in shoots, the authors hypothesized that D15 could inhibit a root-specific CCD activity. However, it is also possible that D15 could be inhibiting the same CCD activity in both roots and shoots. This is because if D15 is a competitive inhibitor for carotenoids binding to CCD enzymes, it is conceivably more effective in roots that contain low levels of carotenoids than in shoots where abundant carotenoid accumulation is found. Understanding the mechanistic basis of D15 inhibition of CCD activities will help discern these possibilities. On another note, it will be interesting to evaluate the impact of overexpressing a root-specific *CCD1* or *CCD4* on LR capacity in the future.

## Carotenoid-derived Signals in Chloroplast Biogenesis, Leaf Development, and Stress Response

While enzymatic cleavage by NCEDs or CCDs is necessary for production of several apocarotenoid signals (e.g., ABA and SLs), an exciting new line of research by the Havaux group indicated that chemical oxidation of β-carotene by singlet oxygen (^1^O_2_) can result in a series of short chain, cleavage derivatives of β-carotene, which are collectively designated carotenoid reactive electrophile species (RES) [e.g., β-cyclocitral (β-CC, C_7_) and dihydroactinidiolide (dhA, C_11_)] (Ramel et al., 2012a,b, 2013a,b,c; Havaux, 2014; Shumbe et al., 2014). In *Arabidopsis* plants exposed to high light, oxidative cleavage of β-carotene by ^1^O_2_ produces β-CC and dhA, which elicit ^1^O_2_ responsive gene expression in the nucleus via a retrograde signaling mechanism and subsequently lead to high light acclimation of the plants (Ramel et al., 2012b; Shumbe et al., 2014). Based on the highly specific impact of carotenoid RES on the ^1^O_2_, but not the H_2_O_2_ marker gene expression, it was suggested that they facilitate plant acclimation to high light stress via the ^1^O_2_ signaling pathway and is independent of H_2_O_2_. Carotenoid RES regulation also differs from the EXECUTER1 protein mediated programmed cell death response to ^1^O_2_ as the *executer1* mutant plants could still acclimate to high light stress and respond to β-CC treatment in a similar way as wild type *Arabidopsis* (Ramel et al., 2012b). Interestingly, high light stress acclimation in the *Arabidopsis* chlorophyll *b* deficient mutant *ch1* and the double mutant between *ch1* and *dde2* [a mutant deficient in jasmonate (JA)] is accompanied by a suppression of JA biosynthesis and decreased JA accumulation in leaves, suggesting an interaction between the carotenoid RES induced ^1^O_2_ response and JA signaling pathways (Ramel et al., 2012b, 2013a,b). It further suggests that high light acclimated plants may be somewhat compromised for JA-mediated responses to pathogens and herbivores. Since ^1^O_2_ could also be generated in plant defense against microbial pathogens (Triantaphylidès and Havaux, 2009), it will be informative to explore whether carotenoid RES signals also directly participate in biotic stress responses by plant hosts.

Besides the carotenoid RES control of nuclear gene expression, involvement of ζ-carotene desaturase (ZDS) precursors in retrograde signaling was also proposed upon characterization of an *Arabidopsis* mutant of *ZDS* (Dong et al., 2007). After a close examination of null *Arabidopsis zds*/*chloroplast biogenesis5* (*clb5*) mutant alleles, abnormal leaf development and cell differentiation as well as compromised auxin responses were also apparent in *clb5*, in addition to the albino phenotype common to mutants that lack a functional ZDS enzyme (Avendaño-Vázquez et al., 2014). Consistent with the report by Dong et al. (2007), the *clb5* mutation modified expression of plastid- and nuclear-encoded genes involved in carotenoid biosynthesis and chloroplast development (Avendaño-Vázquez et al., 2014). Application of an inhibitor of the PDS activity fluridone or introduction of the *pds3* mutation rescued the *clb5* mutant gene expression and leaf development phenotypes, suggesting that products of the PDS reaction and precursors of the ZDS reaction, including di-*cis*-phytofluene and ζ-carotene isomers (ζ-carotene isomerase/Z-ISO catalyzes the isomerization of tri-*cis*-ζ-carotene to di-*cis*-ζ-carotene), may be substrates for the underlying signals regulating chloroplast biogenesis and leaf development (Avendaño-Vázquez et al., 2014; **Figure 1**). Moreover, cleaved signals could be generated from di-*cis*-phytofluene and/or ζ-carotene isomers by CCD4 as *ccd4* mutation in the *clb5* mutant background was able to rescue the *clb5* mutant leaf phenotypes. Exogenous application of ABA or SLs did not affect the appearance of *clb5* leaves, providing further support for novel carotenoid-derived signals controlling chloroplast and leaf development (Avendaño-Vázquez et al., 2014). It remains to be investigated whether these signals are generated locally in leaves or they could be mobile signals transported from roots.

## Perspectives

Overall, substantial progress has been made on identification and characterization of carotenoid-derived signaling molecules in plants. With the exception of ABA, SLs, β-CC, and dhA, a primary unaddressed question regarding these carotenoid-derived signals is their chemical identities. Aside from structure elucidation, there are several additional questions that remain to be answered: (1) How are the structurally uncharacterized apocarotenoid signals produced? Biochemical and genetic analyses suggested that carotenoid precursors could be subjected to catalysis by carotenoid cleavage enzymes. However, direct evidence for involvement of a single or multiple CCD enzymes is still lacking. (2) How are the carotenoid-derived signals transported between different cell types, tissues, and organs? (3) Thus far, work on carotenoids-derived signals, other than ABA and SLs, has been mainly conducted in *Arabidopsis*. Do these signals perform similar functions in other plants? (4) Do overlapping activities or antagonistic effects exist among different carotenoid-derived signaling molecules? (5) Are there additional carotenoid-derived signals remaining to be discovered?

## Conflict of Interest Statement

The author declares that the research was conducted in the absence of any commercial or financial relationships that could be construed as a potential conflict of interest.
